# Electronic Structure of Monolayer FeSe on Si(001) from First Principles

**DOI:** 10.3390/nano12020270

**Published:** 2022-01-14

**Authors:** Karel Carva, Petru Vlaic, Jan Honolka

**Affiliations:** 1 Department of Condensed Matter Physics, Faculty of Mathematics and Physics, Charles University, Ke Karlovu 5, 12116 Prague 2, Czech Republic; 2 Molecular Sciences Department, University of Medicine and Pharmacy “Iuliu Hatieganu”, 400023 Cluj-Napoca, Romania; vlaic_pc@yahoo.com; 3 Institute of Physics, Academy of Sciences of the Czech Republic, Na Slovance 2, 18221 Prague 8, Czech Republic; honolka@fzu.cz

**Keywords:** high-Tc Fe-based superconductivity, interface effects, first-principles calculations

## Abstract

The huge increase in the superconducting transition temperature of FeSe induced by an interface to SrTiO_3_ remains unexplained to date. However, there are numerous indications of the critical importance of specific features of the FeSe band topology in the vicinity of the Fermi surface. Here, we explore how the electronic structure of FeSe changes when located on another lattice matched substrate, namely a Si(001) surface, by first-principles calculations based on the density functional theory. We study non-magnetic (NM) and checkerboard anti-ferromagnetic (AFM) magnetic orders in FeSe and determine which interface arrangement is preferred. Our calculations reveal interesting effects of Si proximity on the FeSe band structure. Bands corresponding to hole pockets at the Γ point in NM FeSe are generally pushed down below the Fermi level, except for one band responsible for a small remaining hole pocket. Bands forming electron pockets centered at the M point of the Brillouin zone become less dispersive, and one of them is strongly hybridized with Si. We explain these changes by a redistribution of electrons between different Fe 3d orbitals rather than charge transfer to/from Si, and we also notice an associated loss of degeneracy between $d_{xz}$ and $d_{yz}$ orbitals.

## 1. Introduction

Superconductivity (SC) has been found in Fe-based compounds with a quasi-2D structure about a decade and half ago [1]. An example with a particularly simple structure is the tetragonal phase of FeSe possessing a critical temperature $T_{c}=8$ K at an ambient pressure [2]. The physics of unconventional SC in bulk Fe-chalcogenides is still unknown. Interestingly, the critical temperature has been shown to increase dramatically when FeSe with a nanoscale thickness is situated on oxide substrates, up to 100 K when the system becomes truly 2D, for monolayer FeSe on SrTiO_3_ substrate [3]. Superconducting circuits represent key components for the advancement of quantum computers [4,5]. These are currently operated at temperatures close to absolute zero, but with the help of high-$T_{c}$ superconductors, the temperature range for quantum circuitry can be increased [6].

The Fermi surface (FS) of bulk FeSe indicates nesting between hole pockets at Γ and electron pockets near M points [7]. However, the electronic structure of FeSe on SrTiO_3_ undergoes a Lifshitz transition, during which the hole pockets at Γ vanish [1]. It was found that for FeSe on SrTiO_3_, an even higher $T_{C}$ can be achieved by the deposition of potassium atoms onto FeSe, which leads to electron doping [8]. This increase in $T_{C}$ is associated to another Lifshitz transition, where electron pockets appear at Γ [9]. Connections between Lifshitz transitions and an increase in $T_{C}$ have already been discovered for other Fe-based superconductors [10]. Clearly, SC in these compounds strongly depends on the detailed orbital ordering and band fillings at the Fermi surface, and information about the change of electronic structure after modifications of various kinds could help to predict whether superconducting temperatures may increase or not [11].

Calculations of FeSe on SrTiO_3_ have found the collinear AFM order diagonally striped along the unit cell to be most favorable [12], similar to the case of ideal bulk FeSe [13]. However, the electronic structure and FS calculated for the checkerboard AFM solution of an isolated monolayer (ML) FeSe [14] appears to conform much better with experimental data for FeSe on top of SrTiO_3_. Simulations performed for FeSe monolayers placed on top of SrTiO_3_ reveal a considerable charge transfer between FeSe and SrTiO_3_; nevertheless, the conclusions about the similarity of the checkerboard AFM solution to the experimental FS remain valid [15]. When the effects of electron doping and substrate-induced phonons are taken into account, the energy difference between collinear and checkerboard AFM orders is drastically reduced, which leads to magnetic frustration [16]. Overall, it is expected that the superconducting state is very close to a magnetic instability, where large spin fluctuations could be mediating the SC [17]. Experimentally, AFM ordering has been confirmed in 1ML FeSe on SrTiO_3_ in the non-superconducting state before electron doping [18]. Therefore, a lot could be learned about superconducting properties of these systems from their magnetic ground states.

The physical mechanism of FeSe SC at the interface to non-conductive perovskite-type oxide substrates is under intense debate, but only a few other substrates have been studied for comparison. In this work, we study the interface between FeSe and semiconducting Si(001) by ab initio DFT methods. Similar to SrTiO_3_, Si(001) has an in-plane lattice constant, which is comparable to that of FeSe (mismatch 1%). Thus, it might allow large-scale single-crystalline interface growth without the formation of azimuthally rotated FeSe domains, as observed on unmatched substrates [19,20]. Compared to SrTiO_3_, Si(001) surfaces are expected to be more reactive due to their dangling bonds. Less tendency for ionic bonding can be expected, which should strongly affect interface charge transfer effects and, therefore, the FS character and magnetism in FeSe monolayers.

We examine here the electronic structure of FeSe monolayers interfaced to Si with special attention on features considered important for supeconductivity. We show changes in the energetical distribution of individual bands that can be tracked in atom-resolved densities of states as well as in occupations of individual Fe 3d orbitals. For the above discussed reasons, we include both the NM state and the checkerboard AFM magnetic ordering.

## 2. Methods

Density funtional theory (DFT) calculations employed the full-potential linear augmented plane wave (FP-LAPW) method, as implemented in the band structure program ELK [21]. The generalized gradient approximation (GGA) parametrized by Perdew–Burke–Ernzerhof [22] has been used to determine the exchange-correlation potential. Spin-orbit coupling (SOC) is known to play key role in the FeSe electronic structure near the Fermi level [23,24] and has been included in the calculation. DFT simulations utilizing GGA-PBE have already succesfully described entirely novel 2D compound binaries exhibiting a similar level of complexity [25], including cases with a strong SOC effect [26]. Calculations for the NM state assumed zero spin-polarization for all atoms, while AFM calculations were started with individual Fe atoms being spin-polarized as in the bulk Fe, and the final momentum on each atom has been achieved self-consistently.

The FeSe adlayer on Si has been modeled using 8 Si(001) layers. Within the supercell, a 8Å thick vacuum spacer was included to simulate the surface. The full Brillouin zone has been sampled by 10 × 10 × 1 k-points. For each magnetic and interface configuration, we have found the optimal distance $d_{\mathrm{Si}-\mathrm{Se}}$ between the interfacial Si plane and its neighboring Se plane with respect to the total energy. The results were compared to the free-standing FeSe monolayer serving as a special idealized limiting case in order to elucidate which properties originate from the Si interface and which are due to the FeSe thickness reduction down to the monolayer limit. The free-standing monolayer is not expected to be stable according to DFT calculations [27].

In our bandstructure plots, we show only bands with a significant projection onto FeSe atomic orbitals. Fe and two non-equivalent Se atoms are distinguished by colors, while the respective color intensity indicates the amount of projection to these atoms.

Notably, the presence of FeSe on Si(001) also leads to a small straining as compared to the bulk FeSe case. In our calculation, the FeSe lattice constant changed to that of Si, a=3.85 Å, which means it grew by 2.5% (slightly less than in the case of SrTiO_3_).

## 3. Results

**Figure 1:**
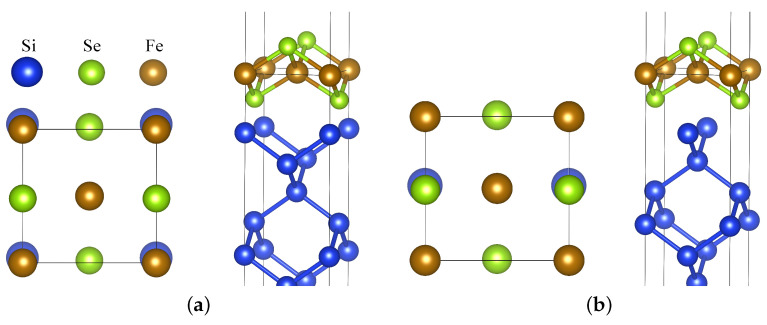
Atomic structure of an FeSe monolayer on the Si(001) surface with two possible interface configurations: (**a**) IC1 and (**b**) IC2. For both, the top and side views are shown. Top view includes Si atoms from the interfacial layer plus Fe and Se atoms. Blue atoms (without label) correspond to Si. Images were drawn using the 3D visualization program for structural models, VESTA [29,30].

There are two plausible relative arrangements of the Si surface with regards to the FeSe ML, denoted as interface configurations IC1 and IC2 (see Figure 1). For IC1, interfacial Se are placed at sites where the next Si would be present if its lattice was continued. Within IC2, Se is placed similarly to if there was another FeSe layer with neighboring Se atoms located where topmost Si atoms are. IC1 is energetically more favorable for both NM and AFM cases according to our calculations. IC1 introduces a larger difference in the environment of the two Fe atom sublattices, possibly resulting in a difference between the corresponding magnetic moments. Notably, a similar situation occurs if a Ti*x*O_2_ interlayer is formed between SrTiO_3_ and FeSe [28].

For AFM FeSe, the spin moment on Fe is 2.09 *μ*B in the case of stand-alone ML. When brought into contact with Si, the moments on the two Fe sublattices become slightly different: for IC1, these are 2.01 and 2.13 *μ*B; for IC2, the difference from 2.09 *μ*B is less than 0.01 *μ*B. Note that FM bulk FeSe DFT calculations converge to an spin-unpolarized state (at each atom) even though the initial Fe potential was spin-polarized. One of the key parameters affecting the electronic structure of FeSe adlayers is the distance from the substrate. The optimal distance differs between the studied cases; it is always larger for AFM compared to NM cases and IC2 compared to IC1 configurations. In all cases, the distance was smaller than the value found for the SrTiO_3_ interface [16], probably due to the low atomic density of the Si lattice and the tendency of Si to form covalent bonds. For NM IC1, we obtain the shortest interplane distance $d_{\mathrm{Si}-\mathrm{Se}}=1.58\;Å$, which is slightly larger than the chalcogen plane distance from the Fe plane. This corresponds to the distance 2.49Å between the nearest Si and Se atoms, which is close to the typical bond length between these atoms in SiSe systems [31].

**Figure 2:**
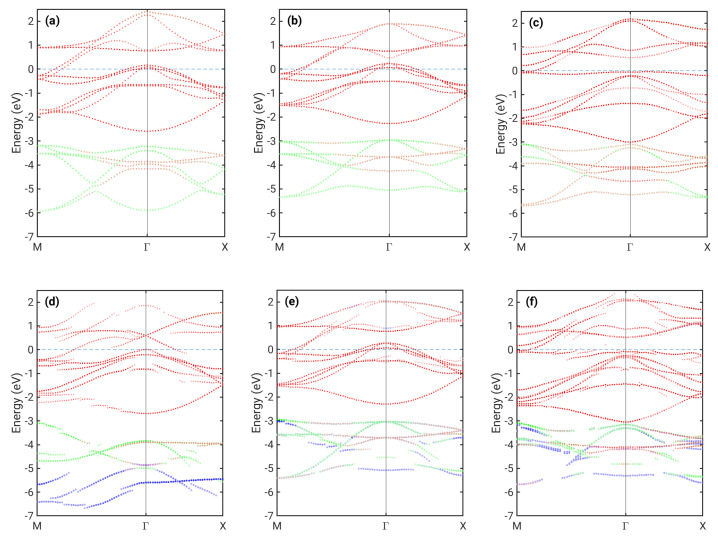
Bandstructure cuts of (**a**) bulk NM FeSe, (**b**) free–standing NM FeSe ML, (**c**) free–standing AFM FeSe ML, (**d**) 1ML NM FeSe on top of Si, IC1, (**e**) 1ML NM FeSe on top of Si, IC2, and (**f**) 1ML AFM FeSe on top of Si. Colors indicate the atomic character as follows: red (Fe), blue (Se in the plane closer to the surface), and green (Se in the plane closer to Si). The composition of colors due to mixed character at individual k-points leads to other color tones seen above, e.g., orange-like.

Let us first review the difference between the calculated in-plane band structure for NM monolayer FeSe and bulk FeSe (Figure 2a,b). In both cases, similar hole pockets near Γ and electron pockets near M are present with only quantitative differences between them, which is in approximate agreement with previous calculations [32]. The picture changes significantly for checkerboard AFM ordering (Figure 2c). Here, a pronounced flat band just below the Fermi level appears, and the hole pocket at Γ shifts below the Fermi level, as already shown for a 1 ML FeSe stand-alone structure [14].

**Figure 3:**
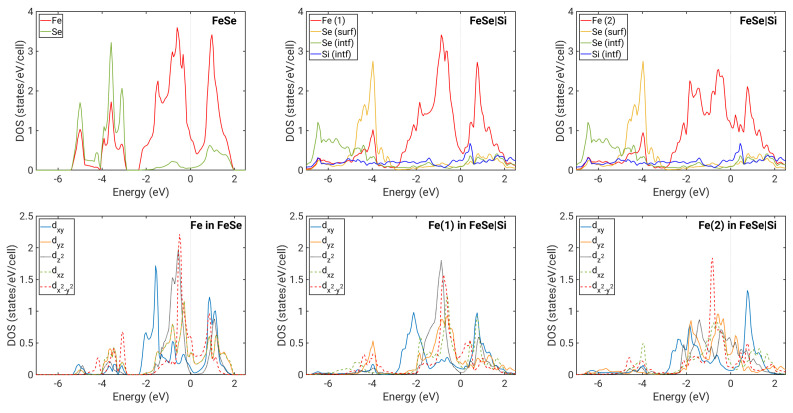
Densities of states. Upper panels: atomic resolved DOSes; lower panels: contributions to DOSes from separate Fe *d* orbitals. Left column of panels: shown for free-standing 1ML NM FeSe. Middle column of panels: shown for 1ML NM FeSe on top of Si (IC1) with contributions from the Fe(1) sublattice. Right column of panels: shown for 1ML NM FeSe on top of Si (IC1) with contributions from the Fe(2) sublattice.

For FeSe on Si (Figure 2d–f), we see a more complex situation. There are effects due to hybridization with Si interfacial bands indicated by the loss of Fe/Se character at some points of the Brillouin zone. When this character is further reduced, the band becomes predominatly Si-type. Si bands are not shown in Figure 2; hence, such a band appears to be discontinuous. Hybridization significantly affects most Se bands. The flat band present in the AFM case just below the Fermi level (Figure 2f) is clearly also highly volatile to any interaction and strongly hybridized to Si bands. Furthermore, contributions from Se atoms in the plane closer to the surface and at the interface became unequal, with the latter occupying predominantly states further below the Fermi level, as is seen more clearly in their DOS (Figure 3).

In the case of the energetically most favorable NM IC1 configuration, the hybridization is stronger due to the smaller distance between FeSe and the substrate. In particular, the Fe band located below −2 eV with an energy minimum at Γ is affected (Figure 2d). Fe in the position above Si atoms within the interface plane in the IC1 configuration (denoted Fe(2)) clearly has a significantly shorter distance to these Si atoms than the Fe(1) located on the other Fe sublattice (Figure 1). Therefore, Fe(2) is affected more by the interaction with Si. Figure 3 shows that the Fe(2) contribution to the DOS differs from that of the free-standing Fe DOS, while Fe(1) is much less modifed. Interfacial Se bands are now significantly broadened, and they provide a large contribution to DOS in energies even below −6 eV, while DOS for the free-standing ML vanishes below ca. −5.5 eV. Furthermore, the DOS at the Fermi level is overall increased for Fe(2).

The Fermi level for the case of the NM IC1 configuration is positioned differently in terms of the bands corresponding to the former hole and electron pockets. By comparing Figure 2b,d, we observe that some bands originally crossing the Fermi level are pushed below it, with only a small hole pocket at Γ remaining. However, our calculations do not show a significant charge transfer between Si and FeSe. In order to understand the shift of the Fermi level relative to different bands, we have also extracted the orbital-resolved DOS for Fe atoms, shown in Figure 3. We first note that for FeSe on Si, the degeneracy between $d_{xz}$ and $d_{yz}$ orbitals is lifted due to the reduced symmetry in the system. Interestingly, this loss of degeneracy has also been observed in bulk FeSe as a consequence of the nematic order [33,34]. The occupations of different orbitals change significantly between the free-standing 1ML NM case and the case of FeSe on Si (IC1). In particular, the occupation of $d_{yz}$ is increased, while that of $d_{xy}$ and $d_{z^{2}}$ is reduced (Table 1). This seems to be connected to the fact that the two FeSe electron pockets located at the M point become flatter when in the IC1 configuration. Furthermore, the outer one of them (mostly of $d_{xy}$ character) is strongly hybridized with Si. The charge from these bands is transferred to hole pockets near Γ, which become almost fully occupied. The small peak in the interfacial Si DOS near −1.5 eV indicates hybridization with Fe states. At approximately the same energy, a peak in the $d_{z^{2}}$ contribution to Fe(2) DOS is present without a counterpart in the free-standing ML. The strong coupling between Fe(2) and Si thus appears to be facilitated predominantly by $d_{z^{2}}$ states, which are extended spatially in the direction of the Fe(2)–Si bond.

**Figure 4:**
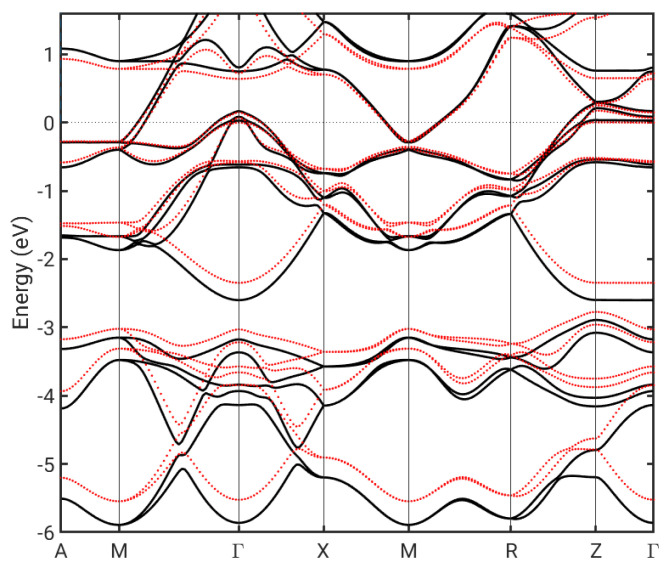
Bulk FeSe bandstructure for the ground state lattice (black lines) and for the lattice stretched to match that of Si (red lines).

Some of the observed differences between the FeSe adlayer on Si and the FeSe bulk solution could originate from the Si-induced straining of the FeSe lattice. Strain has been shown to have an important effect on SC in the case of FeSe on SrTiO_3_ [35], where it leads to an increased Fe-Fe AFM coupling [36]. We have thus examined the effect of Si-induced strain on the bulk band structure, as shown in Figure 4. While some of the bands become less dispersive, the effect in the critical region near the Fermi level does not seem significant.

## 4. Discussion

High $T_{c}$ SC in FeSe monolayers has been associated to the absence of hole pockets around Γ. Hole-like bands were in that case observed slightly below the Fermi level. The so-called incipient bands located up to 100 meV below the Fermi level at Γ may participate in spin fluctuations predicted to lead to a high $T_{c}$ [37]. In our calculations, two of the hole-like FS sheets around Γ present in bulk FeSe are removed under the influence of the Si(001) interface, with only one small hole pocket remaining (Figure 2d). Just a slight electron doping would be needed to shift this hole pocket entirely under the Fermi level, which would lead to a bandstructure potentially consistent with the incipient band scenario. Such doping has been successfully facilitated experimentally by potassium deposition onto FeSe in the case of the SrTiO_3_ interface [8]. In reality, one should also take into account the fact that the result could be affected by Se vacancies, whose role has been found to be highly non-trivial for FeSe on SrTiO_3_ [38].

Our calculations assume a perfect interface between Si and FeSe. This condition may not be necessarily valid for real samples, e.g., due to surface reconstruction effects or intermixing between constituents. However, significant surface reconstructions, e.g., 2 × 1 on Si(001) surface, appear to be improbable. In experiments, interface quality can be partially controlled by various parameters during deposition processes, increasing the proportion of the perfect interface area in real samples. Dynamic stability studies using state-of-the-art DFT methods could help to resolve this problem, but that would go beyond the scope of this work and deserves a dedicated study. Note that FeSe/SrTiO_3_ interfaces had been found to be rather stable, which led to a suggestion of a superlattice based on repetitions of this interface [39].

We stress here that our theoretical contribution on FeSe–Si(001) interfaces is not only interesting from a fundamental physics point of view but may have an impact on future nanotechnological applications or in the area of quantum computing [40]. The Si(100) surface is the most important facet for silicon-based metal–oxide semiconductor device fabrication [41]. Moreover, we want to mention that instead of serving as a substrate, silicon may also become important to terminate and protect FeSe-based heterostructures from deterioration in air: most studies on SC in FeSe/SrTiO_3_ heterostructures use scanning-tunneling spectroscopy and angle-resolved photoemission spectroscopy (ARPES) studies performed under ultra-high vacuum conditions, but FeSe is found to be susceptible to contaminants, such as water, reducing Tc drastically [42].

## 5. Conclusions

We have examined the electronic structure of FeSe on Si(001) for both the NM and checkerboard AFM cases and different interface configurations. For the energetically favorable interface configuration, some of bands originally crossing the Fermi level and forming hole pockets are pushed below it, and the remaining hole pocket around Γ is significantly suppressed. The complete removal of hole pockets from the Fermi surface, as observed for the SrTiO_3_ interface, appears to be possible. Bands corresponding to electron pockets centered at M are flattened, and one of them is strongly hybridized with Si. This is connected to a redistribution of charge between different Fe 3d orbitals, during which the degeneracy between $d_{xz}$ and $d_{yz}$ orbitals is lost. Experimental investigations of the FeSe–Si interface and its critical temperature would allow to test recent intensively discussed theory approaches linked to the interfacial high-Tc superconductivity for an entirely different, non-oxide substrate system. The evaluation of their role could lead to a better understanding of this intriguing phenomenon.

## Figures and Tables

**Table 1:** Occupations of individual Fe 3d orbitals in the different studied NM systems.

	$d_{\mathrm{xy}}$	$d_{\mathrm{xz}}$	$d_{z^{2}}$	$d_{\mathrm{yz}}$	$d_{x^{2}-y^{2}}$
Fe in free-standing FeSe	1.16	1.17	1.39	1.17	1.24
Fe(1) in FeSe on Si, IC1	1.09	1.15	1.32	1.26	1.27
Fe(2) in FeSe on Si, IC1	1.06	1.22	1.25	1.29	1.30

## Data Availability

Data presented in this article are available on request from the corresponding author.

## Abbreviations

The following abbreviations are used in this manuscript:

NM	non-magnetic
AFM	anti-ferromagnetic
2D	two-dimensional
DFT	density functional theory
ML	monolayer
SC	superconductor
GGA	generalized gradient approximation
PBE	Perdew–Burke–Ernzerhof
DOS	density of states
